# Advances in Data-Driven Early Warning Systems for Sepsis Recognition and Intervention in Emergency Care: A Systematic Review of Diagnostic Performance and Clinical Outcomes

**DOI:** 10.7759/cureus.89882

**Published:** 2025-08-12

**Authors:** Abdulkreem Al-Juhani, Rodan Desoky, Ziyad Iskander, Khalid T Alshehri, Abdulaziz A Alshehri, Amaal Almuhaimid, Nouf L Alharbi, Reham H Mominah, Feda M Al-humoud, Abdalrahman Desoky

**Affiliations:** 1 Forensic Medicine, Forensic Medicine Center, Jeddah, SAU; 2 Medicine, College of Medicine, Alfaisal University, Riyadh, SAU; 3 Preventive Medicine, Taibah University, Al-Madinah, SAU; 4 Emergency Medicine, Al Aziziyah Children Hospital, Jeddah, SAU; 5 Medicine, Faculty of Medicine, King Abdulaziz University, Jeddah, SAU; 6 Medicine, Princess Nourah bint Abdulrahman University, Riyadh, SAU; 7 Emergency Medicine, Faculty of Medicine, Almaarefa University, Riyadh, SAU; 8 Emergency Medicine, Almaarefa University, Riyadh, SAU

**Keywords:** artificial intelligence, clinical decision support, diagnostic accuracy, early warning systems, electronic health records, emergency department, machine learning, real-time monitoring, sepsis, sepsis prediction

## Abstract

Sepsis is a life-threatening condition, and early recognition in the emergency department (ED) is crucial for reducing mortality. However, traditional ED sepsis screening tools (e.g., Systemic Inflammatory Response Syndrome (SIRS) and quick Sequential Organ Failure Assessment (qSOFA)) often lack sensitivity for early detection. Machine learning (ML)-based early warning systems have been proposed to address this gap by analyzing complex clinical data in real time. To address this, we systematically reviewed studies (2015-2025) assessing ML-based sepsis warning systems in adult EDs. Databases searched included PubMed, Embase, Scopus, and Web of Science. Included studies reported diagnostic performance and/or clinical outcomes. Overall, a total of 11 studies (primarily retrospective, with a few prospective) were included. ML models using vital signs, laboratory results, and electronic health record (EHR) data demonstrated high discrimination for sepsis, with area under the receiver operating characteristic curve (AUROC) values often exceeding 0.80, and outperformed traditional scoring tools. Many provided earlier warnings, often two to four hours before sepsis was clinically recognized. Implementation studies showed that ML-based alerts expedited treatment; one multi-center system reduced time to antibiotics by ~1.8 hours when alerts were promptly addressed. Some reports noted reduced organ failure and mortality. However, evidence of improved patient outcomes remains inconsistent, likely due to study heterogeneity and limited prospective validation. ML-based early warning systems show strong potential for improving sepsis recognition and treatment in EDs. Further multi-center trials are needed to confirm their impact on outcomes and guide safe implementation.

## Introduction and background

Sepsis remains a major cause of morbidity and mortality worldwide despite advancements in treatment. Timely identification and vigorous intervention for sepsis (e.g., antibiotics and fluid resuscitation within the initial hours) are essential to enhance patient outcomes [1]. Emergency departments (EDs) frequently serve as the initial point for sepsis detection; yet, prompt diagnosis remains challenging due to variable and nonspecific patient presentations [2,3]. 

Various ML-based sepsis prediction models have been developed for implementation in the ED to enhance early detection [4]. These algorithms utilize extensive electronic health record data-comprising vital signs, laboratory results, and clinical notes-to identify sepsis risk factors potentially earlier than clinicians. Numerous studies indicate that ML algorithms can surpass conventional sepsis detection instruments in the ED. A retrospective study's random forest model attained an area under the receiver operating characteristic curve (AUROC) of 0.93, significantly surpassing the AUROC of qSOFA or SIRS within the same cohort [4]. Other academics have suggested ML solutions to mitigate recognized problems such as diagnostic delays and false alarms. Prasad et al. [5] developed an ED sepsis model that eliminates dependence on post-evaluation laboratory tests, hence reducing "diagnostic suspicion" bias, by utilizing just data accessible at triage. Boussina et al. [6] created a deep learning-based early warning system specifically designed to minimize false positives by categorizing doubtful circumstances as indeterminate instead of issuing an alarm. Besides retrospective assessments, certain ML sepsis warning systems have been prospectively evaluated in clinical environments. A multi-hospital investigation of the Targeted Real-Time Early Warning System (TREWS) revealed that an ML-driven warning may detect sepsis cases earlier and was linked to enhanced patient outcomes when doctors responded to the alerts [7]. A further study utilized NLP during triage to forecast sepsis, attaining a high accuracy (AUROC ~0.94) by analyzing nursing triage notes in conjunction with basic vital signs [8]. A recent randomized controlled experiment in an ED demonstrated that the implementation of a real-time ML sepsis warning dramatically enhanced the percentage of patients receiving antibiotics within one hour of triage (68% vs 60% with standard care), hence expediting essential treatment delivery [9]. Notwithstanding these advancements, obstacles persist. The performance of the model may deteriorate when utilized in different patient demographics or hospital environments. Jiang et al. [10] noted that the AUROC of their "qSepsis" model decreased from 0.86 in the development hospital to 0.76 in an external validation cohort, underscoring the necessity of local validation and calibration of ML technologies. Conversely, advanced deep learning models have demonstrated the ability to identify sepsis several hours before clinical acknowledgment - Bedoya et al. [11] reported an ML model that could forecast sepsis a median of 5 hours in advance, significantly surpassing traditional scoring systems such as SIRS or qSOFA. These findings highlight the potential and existing limits of ML-based early warning systems for sepsis. A rigorous assessment of the research is necessary to assess the overall efficacy and real-world impact of various ML technologies in the ED context.

Methodology 

Search Strategy

We performed an extensive literature review to locate publications on ML early warning systems for sepsis in the ED. The subsequent databases were queried: Web of Science, Embase, PubMed (MEDLINE), Scopus, and IEEE Xplore databases. The search covered papers published from January 2015 to June 2025 to capture the most recent decade of research in this rapidly advancing field.

We formulated the search queries utilizing terms from the PICO (Population, Intervention, Comparator, Outcome) framework and integrated them with Boolean operators (AND/OR) to enhance sensitivity. Particularly, the search phrases encompassed variations and synonyms of: Population/Setting: "emergency department," "ED," "emergency room" (to focus on studies in adult emergency care environments). Condition: "sepsis," "septicemia," "severe sepsis," "septic shock" (to encompass all variants of sepsis at risk). Intervention: "machine learning," "artificial intelligence," "ML," "deep learning," "early warning system," "prediction model," "algorithm" (to encompass data-driven predictive systems for sepsis). Data Source: "electronic health records," "electronic medical records," "EHR," "EMR," "real-time data" (emphasizing studies utilizing real-time EHR data inputs). Comparators/Tools: "usual care," "standard care," "NEWS" (National Early Warning Score), "qSOFA" (quick Sequential Organ Failure Assessment), "SIRS" (Systemic Inflammatory Response Syndrome) (to identify research referencing or comparing against standard screening instruments). The phrases were amalgamated utilizing Boolean logic. For instance, one search query was: ("sepsis" OR "septic shock") AND ("emergency department" OR "ED") AND ("machine learning" OR "early warning system") AND ("electronic health record" OR "EHR"). We implemented filters to encompass only research published in English (due to resource constraints for translation) and within the 2015-2025 period. To guarantee comprehensiveness, we also conducted a human review of the reference lists of all included papers for any pertinent studies that the database searches may have overlooked. This systematic search technique was designed with contributions from an information specialist and adheres to Preferred Reporting Items for Systematic Reviews and Meta-Analyses (PRISMA) 2020 principles to guarantee transparency and repeatability. All search queries and results were documented for the reporting of the review. Further information about the search strategy is given in Table 1.

**Table 1:** Search strategy. CINAHL, Cumulative Index to Nursing and Allied Health Literature

PICO component	Search terms/keywords
Population	“emergency department”, “ED”, “emergency room”, “adult emergency care”
Condition	“sepsis”, “septicemia”, “severe sepsis”, “septic shock”
Intervention	“machine learning”, “ML”, “artificial intelligence”, “deep learning”, “early warning system”, “prediction model”, “algorithm”
Data Source	“electronic health records”, “electronic medical records”, “EHR”, “EMR”, “real-time data”
Comparator/Tool	“usual care”, “standard care”, “qSOFA”, “SIRS”, “NEWS”, “sepsis scoring systems”
Boolean Logic	(“sepsis” OR “septic shock”) AND (“emergency department” OR “ED”) AND (“machine learning” OR “early warning system”) AND (“electronic health record” OR “EHR”)
Filters Applied	Language: English; Publication Date: January 2015 to June 2025
Databases Searched	PubMed (MEDLINE), Embase, Scopus, Web of Science, CINAHL

Eligibility Criteria

We established inclusion and exclusion criteria in advance, depending on the objectives of the evaluation. Studies were required to meet the following inclusion criteria: focus on adult ED patients (age ≥18) at risk for or suspected of having sepsis; assess an ML-based early warning system or predictive model for sepsis using real-time patient data from electronic health record (EHR); and specifically evaluate models that continuously or contemporaneously process clinical data. Pertinent outcomes included early sepsis diagnosis accuracy (e.g., AUROC), timeliness of antibiotic administration, and other patient outcomes such as mortality or length of hospital stay. We included studies that either compared the performance of the ML tool with standard care or conventional sepsis screening methods (e.g., quick Sequential Organ Failure Assessment (qSOFA) and National Early Warning Score (NEWS)) or provided sufficient data on the ML model’s efficacy; a direct comparator was not required if the study focused on an ML sepsis alert system. Only studies published in peer-reviewed journals, in English, between 2015 and 2025 were included to ensure a minimum standard of quality and comprehensiveness in reporting. Studies that did not meet these criteria were excluded. We excluded articles that did not incorporate an ML model (e.g., those analyzing only traditional scoring systems without an ML component) or did not utilize EHR data (e.g., those relying solely on bedside clinical judgment or genetic data). We also excluded studies focused on pediatric populations (age <18) or conducted in non-ED settings (such as exclusively critical care units), as our focus was on adult ED patients. Studies lacking any data on model performance or clinical outcomes (e.g., those that merely described a model without assessing its accuracy) were also eliminated. In instances where numerous publications addressed the same intervention and patient group, we incorporated the most complete or recent study to prevent double-counting of outcomes. The qualifying criteria were derived from the PICO elements and adjusted through team discussions before the commencement of screening. We did not eliminate research based on the precise definition of sepsis employed (e.g., Sepsis-2 vs. Sepsis-3 criteria); any definition of sepsis or septic shock was accepted due to the developing nature of sepsis criteria. This comprehensive inclusion strategy aimed to encompass all pertinent evidence about ML-based sepsis alarms in emergency treatment.

Study Selection

All search results were transferred into reference management software, where duplicate entries were detected and eliminated. 

After applying the inclusion and exclusion criteria, a total of 11 studies met the eligibility criteria and were included in the final review. We subsequently implemented a two-stage screening procedure, comprising title/abstract screening followed by full-text screening, in accordance with PRISMA recommendations. Two reviewers separately evaluated the titles and abstracts of all retrieved citations for relevance. Studies that evidently failed to satisfy inclusion requirements (such as animal studies, non-ED environments, or unrelated subjects) were eliminated at this juncture. We acquired the complete texts of all remaining possibly pertinent papers, and two reviewers independently evaluated the full texts against the eligibility criteria. Disagreements or conflicts regarding study inclusion between the two reviewers were handled through discussion and consensus. A third senior reviewer was appointed to adjudicate in instances where consensus could not be achieved, albeit this was infrequently required. 

**Figure 1:**
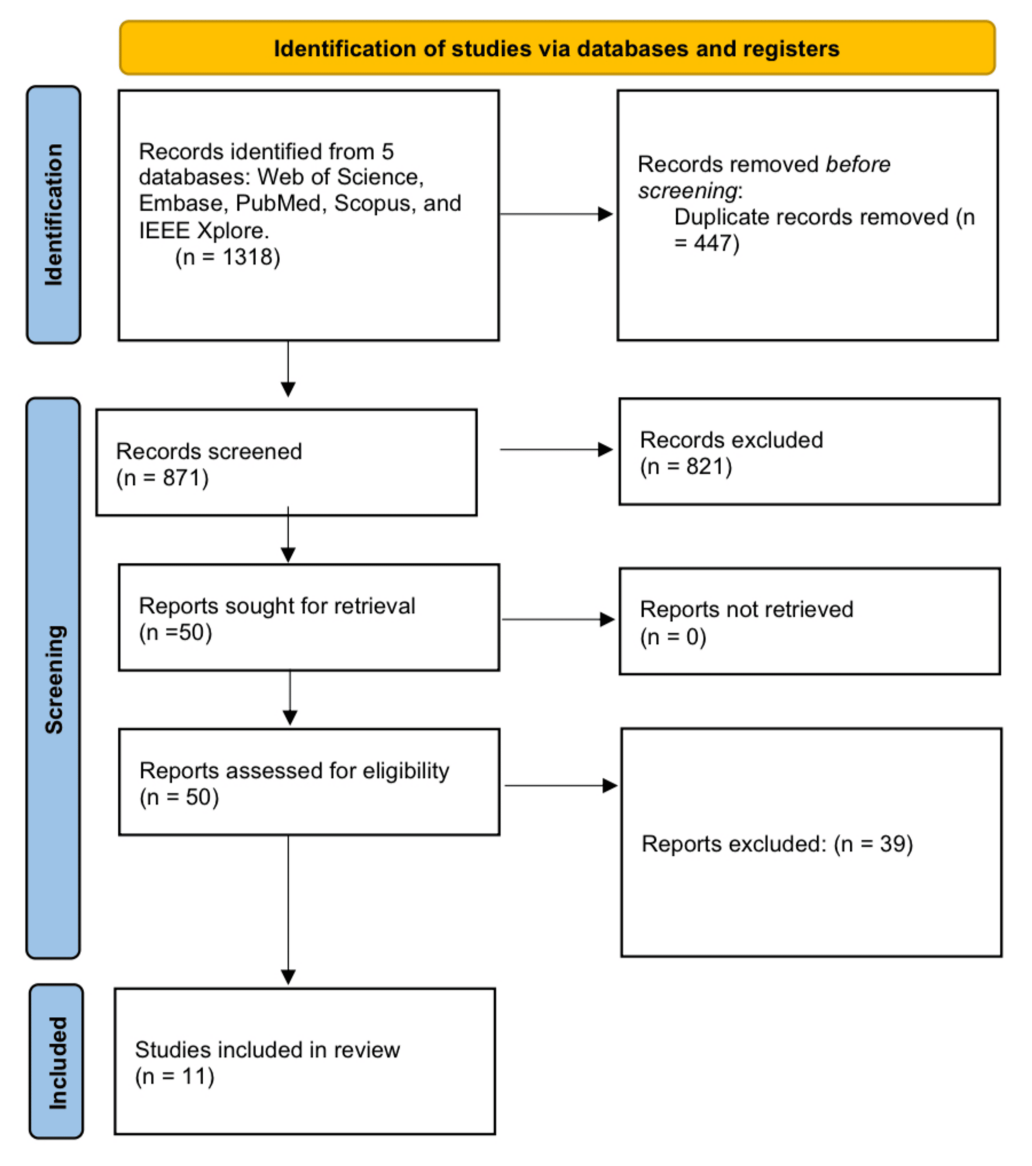
Preferred Reporting Items for Systematic Reviews and Meta-Analyses (PRISMA) flowchart.

The study selection process is illustrated using a PRISMA flow diagram, specifying the number of records identified, screened, excluded, and ultimately included. The PRISMA flowchart depicts the progression of information through the stages of identification, screening, eligibility, and inclusion, as well as the rationale for exclusions at the full-text review stage. This guarantees transparency in the filtering of studies and offers a visual overview of the selection process in accordance with PRISMA 2020 reporting criteria (Figure 1).

Data Extraction and Synthesis

For each study matching the inclusion criteria, we extracted pertinent data utilizing a standardized form specifically developed for this evaluation. Two reviewers conducted separate data extraction, and the results were verified for accuracy. The extracted data comprised: study characteristics (first author, publication year, country), study design (retrospective or prospective cohort, randomized trial, etc.), characteristics of the ED patient population (sample size, inclusion criteria, sepsis definition employed), specifics of the ML intervention (type of algorithm or model, input features from electronic health records, implementation of real-time alerting), details of the comparator or usual care if relevant (e.g., any sepsis screening tool utilized in the control group), and all reported outcomes of interest. Key performance parameters, including sensitivity, specificity, AUROC for sepsis prediction, time to antibiotic delivery, death rates, and hospital length of stay, were documented when accessible. We also recorded whether the study was focused on model development/validation or implementation, specifically indicating if the ML tool was used in practice and influenced clinical care. Discrepancies in data extraction were handled by consensus or third-party adjudication to ensure correctness. 

In light of the expected variability in study designs and outcomes, we want to conduct a narrative synthesis of the results. We will evaluate the performance of several ML models in predicting sepsis, focusing on accuracy and timeliness, and compare them to traditional screening methods (if available), along with their effects on patient outcomes. Should adequate homogenous data be accessible (e.g., numerous studies presenting AUROC for analogous outcomes under similar conditions), we will contemplate a quantitative synthesis. A meta-analysis of diagnostic accuracy, utilizing pooled AUROC or sensitivity/specificity, may be conducted with a random-effects model, adhering to recognized methodologies for the meta-analysis of prediction models or diagnostic tests. Nevertheless, owing to anticipated discrepancies in model types and outcome definitions, a meta-analysis may be unsuitable; in this instance, data will be displayed in tables and figures and summarized qualitatively. All analyses and reporting will comply with the 2020 PRISMA criteria to guarantee clarity and scientific rigor.

Quality Assessment

We evaluated the risk of bias and overall quality of each included study using the Prediction model Risk of Bias Assessment (PROBAST) method. PROBAST is explicitly formulated for assessing research that creates or evaluates prediction models, including ML-based sepsis prediction algorithms, within systematic reviews. The document comprises 20 signaling questions categorized into four principal domains: Participants, Predictors, Outcome, and Analysis, aimed at assessing potential biases in study design, execution, and analysis. Each domain is assessed as exhibiting *low*, *high*, or *unclear* risk of bias, resulting in an overall risk-of-bias evaluation for each study. The tool also tackles issues related to the relevance of the study to the review topic (e.g., if the demographic or environment aligns with our ED context). Two reviewers separately utilized the PROBAST checklist for each included study, resolving discrepancies through discussion. We focused on prevalent sources of bias in prediction model studies, such as the definition and identification of sepsis (Outcome domain), the selection and measurement of predictors, the management of missing data, the risk of model overfitting, and the evaluation of performance on an independent validation set (Analysis domain). The findings of the risk of bias evaluation will be presented in the review, along with a summary of concerns for each domain pertaining to each study. Employing PROBAST in this fashion offers a clear and systematic assessment of study quality, assisting readers in evaluating the robustness of evidence for ML-based sepsis early warning systems. All methodological procedures outlined above were executed in accordance with known systematic review practices and are documented in compliance with PRISMA 2020 to ensure transparency, consistency, and completeness of our methodologies. By meticulously delineating the search strategy, selection criteria, data extraction methodology, and quality assessment techniques, we sought to mitigate bias and augment the dependability of the findings in this systematic review (Table 2).

**Table 2:** Prediction model Risk-of-Bias Assessment (PROBAST).

Study	Participants	Predictors	Outcome	Analysis
Horng et al. (2017) [1]	Low	Low	Unclear	Low
Wardi et al. (2021) [2]	Low	Unclear	Low	Unclear
Lin et al. (2021) [3]	Low	Unclear	Low	Low
Kijpaisalratana et al. (2022) [4]	Low	Low	Unclear	Unclear
Prasad et al. (2023) [5]	High	Low	Low	Low
Boussina et al. (2023) [6]	Low	Low	Low	High
Adams et al. (2022) [7]	Low	Low	Low	High
Brann et al. (2024) [8]	Low	Low	Low	Low
Kijpaisalratana et al. (2024) [9]	Low	Low	Low	Low
Jiang et al. (2025) [10]	Low	Low	Low	Low
Bedoya et al. (2020) [11]	Low	Low	Low	Low

Results

Study and ML Model Characteristics

Eleven studies fulfilled the inclusion criteria, encompassing various geographic locations and ED environments. The characteristics of the included studies, including country, ED setting, sample size, sepsis definition, and inclusion criteria, are summarized in Table 3. The majority (7 of 11) were executed in the United States, predominantly in large university or healthcare system EDs. The remaining individuals were from Asia (Thailand, Taiwan, and China) and were often affiliated with tertiary-care hospital EDs. The study designs primarily consisted of retrospective cohort or observational assessments of ED contacts. A multi-center study prospectively assessed an established sepsis alarm across five hospitals, while another study employed a cluster-randomized design to compare an ML-driven alert with standard care in a single ED. Sample sizes exhibited considerable variability, ranging from fewer than 2,500 patients in model development cohorts to over 1,000,000 ED visits in extensive health system datasets, illustrating the heterogeneity of data sources. Numerous studies delineated sepsis outcomes utilizing standard clinical criteria, with seven utilizing or citing the Sepsis-3 consensus definitions (suspected infection coupled with acute organ dysfunction). Several utilized antiquated Sepsis-2 definitions predicated on Systemic Inflammatory Response Syndrome (SIRS) criteria or pragmatic proxies derived from clinical evidence. For instance, one study classified sepsis cases using International Classification of Diseases (ICD) diagnosis codes for infection at ED discharge, while another utilized ICD-10 sepsis codes indicating infection with organ dysfunction. The studied patient populations were solely adults (age ≥18 years) presenting to the ED with possible infection or sepsis. Certain studies included all patients presenting to the ED, while others enhanced the sample with confirmed sepsis cases and controls for model training. The prevalence of sepsis in ED cohorts varied between around 3% and 30%, contingent upon case definitions and inclusion criteria. 

**Table 3:** Study characteristics and patient inclusion. CMS, Centers for Medicare and Medicaid Services; ICD, International Classification of diseases; EHR, electronic health records; SIRS, systemic inflammatory response syndrome; MGH, Massachusetts General Hospital; UCSD, University of California San Diego; ML, machine learning; TREWS, temporal reasoning early warning system; MIMIC-IV, Medical Information Mart for Intensive Care version 4; ICU, intensive care unit

Study (Author, year)	Country/setting	Study design	Sample size	Patient population	Inclusion criteria	Sepsis definition used
Horng et al. (2017) [1]	United States - Single tertiary academic ED (Boston)	Retrospective observational	N = 230,936 ED visits	All ED patients (adults) at one hospital	All consecutive ED visits 2008-2013 (no exclusions)	“Infection diagnosed in ED” (infection-related ICD-9 diagnosis at discharge)
Wardi et al. (2021) [2]	United States - Two academic EDs (California and Georgia)	Retrospective cohort (multi-site)	~180,000 ED patients (2014-2019)	Adult ED patients at two centers	ED patients meeting severe sepsis criteria during the study period	Severe sepsis per CMS criteria (culture + antibiotics + ≥2 SIRS + organ dysfunction within 6h); also analyzed Sepsis-3 for sensitivity
Lin et al. (2021) [3]	Taiwan - Two hospitals (academic EDs)	Retrospective development and external validation	Dev: 8296 patients (21% sepsis); Ext: 1744 patients (29% sepsis)	Adult ED patients at 2 separate institutes	All ED encounters in two hospitals (different care levels) were used for model development and external test.	Sepsis-3 consensus criteria (suspected infection + acute organ dysfunction) used as reference standard.
Kijpaisalratana et al. (2022) [4]	Thailand - Single tertiary ED (Bangkok)	Retrospective single-center	(Not reported; single-ED cohort)	Adult ED patients at one academic hospital	All ED presentations considered; outcome labeled by final sepsis diagnosis.	ICD-10-CM sepsis diagnosis code as outcome label (presumed infection with organ dysfunction)
Prasad et al. (2023) [5]	United States - Single academic ED + multi-ED validation	Retrospective cohort with external validation	Dev: 1,663 patients; Val: 784 patients	Adult ED patients (sepsis cases and controls) at MGH; external cohort from 4 EDs	Adult ED encounters 2014-2016 (development) and 2016-2018 (validation); included sepsis cases and non-sepsis controls.	Sepsis per Rhee criteria (suspected infection + ≥1 organ dysfunction)
Boussina et al. (2023) [6]	United States - Two EDs in one health system (California)	Quasi-experimental (before-after)	N = 6217 sepsis patients (5065 pre-implementation; 1152 post)	Adult ED patients with sepsis in UCSD Health (2 hospitals)	ED encounters 2021-2023 meeting sepsis criteria; pre vs. post COMPOSER deployment	Sepsis-3 criteria (suspected infection plus acute organ dysfunction within 12h of ED arrival)
Adams et al. (2022) [7]	United States - Five hospitals (academic + community)	Prospective multi-site cohort	590,736 patients monitored; 6,877 sepsis alerts analyzed	All ED admissions across 5 hospitals (with EHR sepsis alert)	All patients monitored by the TREWS ML alert system; focused on those alerted before antibiotics	Sepsis is defined by alert criteria (ML early warning for suspected sepsis before treatment; aligned with Sepsis-3 clinical criteria)
Brann et al. (2024) [8]	United States - Four academically affiliated EDs	Retrospective multicenter cohort	N = 1,059,386 ED encounters (2015-2021; 3.45% developed sepsis)	Adult ED patients across 4 hospitals	All adult ED visits over the study period after exclusions (pediatric, cardiac arrest, and missing data)	Sepsis-3 criteria (presumed severe infection + acute organ dysfunction) as primary outcome
Kijpaisalratana et al. (2024) [9]	Thailand - Single tertiary ED (Bangkok)	Cluster-randomized trial (real-time alert)	N = not stated (all eligible sepsis cases during trial)	Adult ED patients at one hospital (sepsis alerts vs. standard care)	ED patients meeting sepsis trigger criteria; randomized by time blocks to ML alert vs. usual care	Sepsis-3 criteria for sepsis identification in ED (suspected infection with organ dysfunction) are used for alert triggering
Jiang et al. (2025) [10]	China - Single academic ED (Wuhan) + external US data	Retrospective single-center development and external validation	N = 414,864 ED visits (internal cohort); external validation on MIMIC-IV ED database (US)	Adult ED patients at a tertiary hospital in China	ED visits 2015-2022, age ≥18; excluded cardiac arrest on arrival and incomplete records	Sepsis-3 definition (infection suspicion and ≥2 SOFA rise) for ED sepsis diagnosis
Bedoya et al. (2020) [11]	United States - Single tertiary hospital (North Carolina)	Retrospective cohort (internal and temporal validation)	N = 82,765 hospital encounters (42,979 train + 39,786 temporal test)	Adult admitted patients (ED arrivals) at a large academic center	All adult inpatients (non-ICU and ICU) with potential sepsis, 2014-2015 (train) and 2018 (temporal validation)	Sepsis-2 (≥2 SIRS + blood culture order + ≥1 organ failure) used to identify sepsis cases

The assessed ML models were diverse in type and complexity. Detailed specifications of the ML models-including model type, learning approach, feature sets, and validation strategy-are presented in Table 4. Five studies constructed ensemble tree-based models, including random forests and gradient-boosted decision trees. Two studies employed logistic regression techniques, including a basic L2-regularized logistic model, while an earlier study utilized a support vector machine classifier. Multiple studies examined deep learning methodologies: one utilized a two-layer neural network within a survival analysis paradigm for time-to-event forecasting, while another applied a recurrent neural network (incorporating a multi-output Gaussian process element) to manage time-series electronic health record data. Significantly, a minimum of three models included natural language processing (NLP) of triage text to enhance structured data. For example, free-text triage nurse notes and primary complaints were utilized as supplementary features in certain models, highlighting the significance of unstructured data for early indicators of sepsis. Brann et al. [8] compared an NLP-enhanced model utilizing triage notes to conventional nurse triage screening. Model interpretability exhibited variability. Most methodologies operated as *black box* predictors, offering minimal real-time elucidation to consumers. Two studies explicitly favored simpler, more interpretable models: Prasad et al. [5] chose a parsimonious logistic regression with a limited number of predictors to ensure transparency, while Jiang et al. [10] similarly selected a 12-variable logistic model (*qSOFA-sepsis* or qSepsis) to enable clinicians to examine feature weights. The studies generally verified their models either internally using holdout data or temporally independent test sets, with around half conducting external validations using separate hospital datasets. External validation, when conducted, frequently exhibited a decline in performance relative to development cohorts (as elaborated later), highlighting difficulties in generalizability across locations.

**Table 4:** Machine learning model specifications. AISE, Artificial Intelligence Sepsis Expert; SOFA, Sequential Organ Failure Assessment; CRP, C-reactive protein; BP, blood pressure; HR, heart rate; RR, respiratory rate; Temp, temperature; ESI, Emergency Severity Index; NLP, natural language processing; COMPOSER, conformal prediction-based deep learning model for sepsis alerting; TREWS, Targeted Real-Time Early Warning System; MIMIC-IV, Medical Information Mart for Intensive Care IV database; RNN, recurrent neural network; MGP, multi-output Gaussian process; AUROC, area under the receiver operating characteristic curve; SIRS, Systemic Inflammatory Response Syndrome; qSOFA, Quick Sequential Organ Failure Assessment; MEWS, Modified Early Warning Score; NEWS, National Early Warning Score

Study (author, year)	ML model type	Learning approach	Input features used	Interpretability	External validation	Comparator(s)
Horng et al. (2017) [1]	Support vector machine (SVM); tested text vs. non-text models	Supervised (train/val/test split within single ED data)	Triage vital signs, demographics, chief complaint, triage nurse free-text notes (bag-of-words and topic modeling)	No (black-box SVM; no explainability in use)	No (internal validation only; single center)	Incremental models compared: vitals-only vs. +chief complaint vs. +nurse text (text-based model improved AUC)
Wardi et al. (2021) [2]	Hybrid model: Modified Cox proportional hazards with 2-layer neural network (AISE)	Supervised time-to-event (survival analysis); transfer learning for second site	40 input variables: vital signs, laboratory values, SOFA score components, comorbidities, etc.	No (complex survival neural model; no user-facing explainability)	Yes - validated at the second hospital with model fine-tuning (transfer learning)	Compared to a model trained from scratch at a new site (transfer learning vs. no-transfer), no explicit comparison to qSOFA/SIRS in the paper
Lin et al. (2021) [3]	eXtreme Gradient Boosting (XGBoost) ensemble classifier	Supervised classification (train on the dev hospital, tested on the external hospital)	ED triage vital signs, laboratory tests (e.g., CRP, creatinine, platelets, etc.), demographics; top features included both vital signs and labs	No (feature importance analyzed post-hoc, but model itself not intrinsically interpretable)	Yes - external validation on separate hospital data	Standard ED sepsis screening scores (qSOFA, SIRS) - model outperformed qSOFA (AUC 0.56) and SIRS (0.68)
Kijpaisalratana et al. (2022) [4]	Random Forest (best-performing; also tested logistic regression, neural net, gradient boosting)	Supervised classification (retrospective single-center)	Vital signs (e.g., BP, HR, RR, Temp), demographics, triage acuity (ESI), mode of arrival, free-text chief complaint (NLP)	No (ensemble ML; no mention of explainable output)	No (internal only, no separate external dataset)	Existing sepsis screening tools: SIRS, qSOFA, MEWS - ML model significantly outperformed all (e.g., RF AUC 0.93 vs. SIRS 0.81)
Prasad et al. (2023) [5]	L2-regularized Logistic Regression (two variants: basic vs. with additional queries)	Supervised classification (feature-selected logistic model); included manual review queries for one version	“Bland” model: triage vitals and demographics; Enhanced model: +three yes/no risk questions derived from triage note (via NLP) + past medical history	Yes (parsimonious logistic model; limited features for transparency)	Yes - validated on a four-hospital external cohort	qSOFA (baseline early screening) - investigational models outperformed qSOFA in all cohorts (e.g., AUC 0.78 vs. qSOFA 0.66)
Boussina et al. (2023) [6]	Deep learning model (COMPOSER) with uncertainty flagging (conformal predictions)	Supervised real-time prediction (trained on retrospective data; deployed in EHR)	Continuous EHR data feeds: vital signs, lab results, and other real-time clinical data (streamed; ~50 variables)	No (black-box deep model; designed to flag indeterminate cases rather than explain features)	No standalone external validation (prospective deployment in two EDs of the same health system)	Usual care pre-implementation (no ML alert) - evaluated differences pre vs. post deployment; no direct metric comparison to qSOFA/SIRS in study (focus was on outcomes)
Adams et al. (2022) [7]	Ensemble ML model integrated in EHR (TREWS alert system - proprietary)	Supervised learning (trained on large multicenter data); prospective real-time deployment	EHR-derived features: vital signs, laboratory values, demographics, and clinical observations (continuously monitored)	Partially - alert interface provided relevant patient data; model internals not fully transparent to users	Yes - deployed across 5 diverse hospitals (2 academic, 3 community)	Usual care (no alert) - outcomes compared between alert-acknowledged vs. alert-ignored patients; no direct comparison to legacy scores (study evaluated alert versus no-alert effect)
Brann et al. (2024) [8]	Decision tree-based ensemble (gradient boosted trees) with NLP features	Supervised classification (trained on multicenter data, tested on hold-out set)	Nursing triage free-text notes (NLP vectorization) + triage vital signs, age, sex, triage acuity, etc. (no labs at triage); plus optional lab data in a separate model	No (complex ensemble with text embedding; feature importance examined, but model not intrinsically interpretable)	Yes - internally validated across 4 hospitals (train/test split; results consistent by site)	ED triage nurse sepsis screening (protocol) - ML model identified 76% of sepsis cases missed by nurse-initiated screening and 97.5% of those identified by nurses (outperformed routine triage screening)
Kijpaisalratana et al. (2024) [9]	Random Forest ML-assisted sepsis alert (integrated into EHR workflow)	Supervised model (trained on retrospective ED data) deployed in real-time (prospective trial)	Vital signs, demographics, triage data, and chief complaint text (same feature set as 2022 model) fed into real-time alert system	No (model logic not exposed; alert provided binary trigger to clinicians)	N/A (prospective evaluation in same hospital; no new external site)	Standard sepsis alert protocol (usual care) - compared ML alert vs. no alert in cluster-randomized fashion; also compared performance to SIRS/qSOFA/MEWS (RF AUC ~0.93 vs. SIRS 0.84, qSOFA 0.73)
Jiang et al. (2025) [10]	qSepsis models - tested Logistic Regression (best), Random Forest, XGBoost.	Supervised classification (trained on single-center ED data, tested on external dataset)	Non-lab clinical features only: triage vital signs, demographics, ED observations (no laboratory results)	Yes (final chosen model was a logistic regression with ~12 predictors, facilitating weight inspection)	Yes - externally validated on MIMIC-IV ED database (US ICU/ED records)	SIRS, qSOFA, MEWS - qSepsis LR model outperformed all (external AUROC 0.766 vs. SIRS 0.704, qSOFA 0.579, MEWS 0.600)
Bedoya et al. (2020) [11]	Deep learning: multi-output Gaussian Process + Recurrent Neural Network (MGP-RNN)	Supervised time-series modeling (trained on 2014-2015 data, temporally validated on 2018 data)	Vital signs (time-series), labs, medications administered, comorbidities, demographics - continuously updated during hospitalization	No (complex RNN; authors assessed feature influence offline, but no real-time explanations)	No external site (internal temporal validation 3 years later at the same hospital)	Three ML models (RF, Cox, penalized LR) and three scoring systems (SIRS, qSOFA, NEWS) - MGP-RNN achieved the highest AUROC (0.88) and detected sepsis ~5 hours earlier than onset.

Diagnostic Performance of ML Sepsis Alerts

All ML-based early warning models exhibited at least significant discrimination in diagnosing sepsis in the ED, with the majority reaching robust performance. Key performance metrics of the evaluated models, including AUROC, sensitivity, specificity, and implementation status, are compiled in Table 5. The reported AUROCs during internal validation ranged from approximately 0.78 to 0.94. Basic models utilizing only triage vitals and demographics attained AUROCs in the high 0.70s to low 0.80s, whereas more intricate or complete models (e.g., incorporating laboratory data or text analysis) frequently surpassed 0.85-0.90 in the ED context. An NLP-enhanced gradient boosting model assessed on about 1 million ED visits achieved an AUROC of 0.94 at triage, utilizing solely vital signs and free text. Conversely, a logistic regression model utilizing solely triage vital signs achieved an approximate AUROC of 0.78, which enhanced to around 0.84 upon the inclusion of supplementary risk indicators from triage notes. Multiple models achieved or exceeded an AUROC of 0.90 in their initial analyses, demonstrating exceptional discrimination for early sepsis prediction within the research populations. 

**Table 5:** Model performance and deployment outcomes. AUROC, area under the receiver operating characteristic curve; SVM, support vector machine; ICU, intensive care unit; Youden’s J, Youden’s J statistic; XGBoost, eXtreme Gradient Boosting; RF, random forest; BP, blood pressure; HR, heart rate; RR, respiratory rate; Temp, temperature; ESI, emergency severity index; NLP, natural language processing; COMPOSER, conformal prediction-based deep learning model for sepsis alerting; EHR, electronic health record; TREWS, targeted real-time early warning system; PPV, positive predictive value; NPV, negative predictive value; LOS, length of stay; MIMIC-IV, Medical Information Mart for Intensive Care IV database; LR, logistic regression; MLASA, machine learning-assisted sepsis alert; qSepsis, quick sepsis; ED, emergency department; qSOFA, quick Sequential Organ Failure Assessment; SIRS, Systemic Inflammatory Response Syndrome; MEWS, Modified Early Warning Score

Study (author, year)	AUROC (internal/external)	Sensitivity/specificity	Outcome improvements	Deployment mode	Alert user
Horng et al. (2017) [1]	0.86 internal test AUC (SVM with full text); no external validation	~81% sensitivity (text model in ICU-admitted subset); specificity not reported (threshold optimized by Youden’s J)	Model not deployed; retrospective evaluation only)	Not deployed (retrospective study)	N/A (no live alert)
Wardi et al. (2021) [2]	>0.80 AUROC internal (8-12-hour shock prediction); improved to ~0.82 at external site after transfer learning	Reported as excellent sensitivity after transfer learning (exact values not provided); specificity increased with transfer learning	No patient outcome reported; focus on predictive performance for delayed septic shock	Not deployed (retrospective model development; tested on historical data)	N/A (no live alert)
Lin et al. (2021) [3]	0.86 internal AUC; 0.75 external AUC (XGBoost model)	Sensitivity 87%, specificity 85% (at triage, dev set); external sensitivity lower as performance dropped (not specified)	No clinical deployment; the model showed reduced performance on the external cohort due to case mix	Not deployed (retrospective only; external validation on past data)	N/A (no live alert)
Kijpaisalratana et al. (2022) [4]	~0.93 internal AUC (RF model); no external validation	At the operating threshold, high sensitivity and specificity (~90%+ each) implied by AUC (exact values not given)	No deployment; study showed ML significantly outperformed qSOFA/SIRS in detection accuracy.	Not deployed (retrospective study)	N/A (no live alert)
Prasad et al. (2023) [5]	0.78 AUC (triage data model) vs. 0.84 AUC (with queries) - internal; 0.74-0.79 AUC in 4-hospital external validation	Bland model ~70% sens/spec, Enhanced model ~80% sens/spec (approximate, from AUC ~0.83; exact values not stated)	No direct patient outcomes measured; demonstrated earlier risk detection at triage versus standard screening.	Not deployed (retrospective modeling with post-hoc validation)	N/A (no live alert)
Boussina et al. (2023) [6]	Model AUC ~0.85-0.90 was reported previously; the current study focused on outcomes	Alert threshold set for high sensitivity; exact sens/spec not reported in outcome study.	↓ In-hospital mortality by 1.9% (absolute; 17% relative reduction); ↑ bundle compliance by 5% (absolute); ↓ 72 hours SOFA Δ by 4% after COMPOSER deployment	Real-time EHR deployment (Best Practice Alert pop-up to nurses) in ED workflow	Nurse (primary alert to triage/charge nurses, who then notified clinicians)
Adams et al. (2022) [7]	Model AUC not explicitly in publication; prior reports ~0.85-0.88	Threshold tuned for high NPV; ~82% sensitivity, PPV ~20% per commentary	↓ Mortality by 3.3% (absolute, when providers responded to alert within three hours); ↓ organ failure and LOS in alert-confirmed group; benefits largest in high-risk alert patients	Real-time integrated EHR alert (TREWS) across five hospitals; providers received and acted on alerts prospectively	Clinician (ED physicians prompted to confirm sepsis and start treatment via alert interface)
Brann et al. (2024) [8]	0.94 AUC at ED triage (no labs) internal; 0.97 AUC with labs by 12h; external performance similar by site	Sensitivity 87%, specificity 85% at triage (primary model); NPV ~99%, PPV ~18% (3.45% prevalence)	Not deployed; showed the NLP model could detect 76% of sepsis cases missed by standard triage screening.	Not deployed (retrospective multicenter study)	N/A (model not in clinical use)
Kijpaisalratana et al. (2024) [9]	~0.93 AUC (RF model, as previously validated) in live trial; no external dataset	High sensitivity protocol (alert triggered on sepsis criteria; SIRS sensitivity ~85% as comparator	↑ Timely antibiotics: +8.3% patients received antibiotics <1 hour, +5.5% <3 hours with ML alert; Improved diagnostic precision (fewer false activations vs. SIRS/qSOFA)	Real-time EHR ML alert (MLASA) vs. standard screening in ED (cluster-randomized by shift)	Clinician (ED physicians received automated sepsis alerts within EHR and confirmed treatment)
Jiang et al. (2025) [10]	0.862 AUC internal (qSepsis LR); 0.766 AUC external (MIMIC-IV ED)	At ~85% sensitivity, specificity ~70% externally - trade-off noted by authors	Not deployed; proposed *qSepsis* could enable lab-free early screening - high NPV 0.99, but low PPV ~0.18, leading to false alarms)	Not deployed (retrospective development with external validation)	N/A (no live alert)
Bedoya et al. (2020) [11]	0.88 AUC internal (four hours before sepsis onset); temporal validation similar (model outperformed all comparators)	Sensitivity ~80% at four hours early-warning (est. from median five hours lead time); specificity higher than traditional screens (qSOFA, etc.)	Not deployed in this study; demonstrated five-hour earlier sepsis detection vs. clinical onset	Not deployed (retrospective training; prospective integration reported later)	N/A (model was evaluated offline; later work integrated it into clinical workflow)

When assessed, the external validation performance was typically marginally worse than the internal results, underscoring the significance of local calibration. Lin et al. [3] indicated that the AUROC of their XGBoost model decreased from 0.86 in the development hospital to 0.75 in a different hospital cohort. The qSepsis logistic model by Jiang et al. [10] attained an AUROC of 0.862 on its derivation dataset, but only 0.766 when evaluated on an external ICU/ED database. Wardi et al. [2] demonstrated that performance improved at a secondary site following the implementation of transfer learning (from >0.80 to approximately 0.82 AUROC for early shock prediction), indicating that adaptation to local data can restore accuracy. Despite some performance degradation outside the development context, the ML models sustained accuracy exceeding random chance across many contexts.

The models exhibited a preference for greater sensitivity in sepsis detection at the operating thresholds established by the study authors. Most technologies attained sensitivity between 80% and 90% in detecting sepsis cases in the ED. An NLP-based triage model achieved approximately 87% sensitivity and 85% specificity at ED presentation, whereas a deep learning early warning system was capable of identifying around 80% of sepsis cases roughly four hours before start. Specificities exhibited considerable variability, often ranging from 70% to 90%, contingent upon the threshold and the prioritization of reducing false negatives. Several internally validated models demonstrated a balanced high specificity and sensitivity (about 85%-90% for both); nevertheless, external validations typically revealed modest specificity (e.g., around 70%), whereas sensitivity was approximately 85% in a study by Jiang et al. [10]. In numerous instances, authors deliberately selected threshold criteria to optimize sensitivity: the COMPOSER system by Boussina et al. [6] employed a high-sensitivity alert threshold, and the trial by Kijpaisalratana et al. [9] similarly adopted a *high sensitivity* alert protocol, aiming for approximately 85% sensitivity, comparable to SIRS criteria. A disadvantage of this method was a comparatively low positive predictive value (PPV) for some alarms, often ranging from 15% to 20% PPV in ED populations. For example, the triage NLP model, with a sepsis prevalence of 3.5%, exhibited a PPV of approximately 18% and a negative predictive value of about 99%. In contrast, the TREWS system's alert in a multi-hospital research demonstrated a PPV of around 20% with a sensitivity of approximately 82%. These statistics illustrate the intrinsic trade-off: to identify nearly all sepsis cases promptly, several non-septic patients may activate an alert (resulting in a high false-positive rate), a challenge recognized in the calibration of the systems. 

The ML models consistently surpassed traditional sepsis screening techniques when comparisons were available. In direct comparisons, ML algorithms demonstrated superior discrimination compared to scoring systems such as qSOFA, SIRS, or MEWS. In one study, a random forest model attained an AUROC of approximately 0.93, far surpassing the SIRS (0.81) and qSOFA (0.56) findings for the same cohort. The qSepsis model (logistic regression excluding laboratory data) also surpassed qSOFA and SIRS in external validation (AUROC 0.766 compared to 0.579 for qSOFA). Bedoya et al. [11] discovered that their deep learning model achieved the highest AUROC (0.88) compared to many alternatives and was capable of detecting sepsis far earlier than conventional criteria. These patterns suggest that ML-based warnings, utilizing comprehensive electronic health record data and sophisticated algorithms, can significantly enhance the accuracy of basic rule-based ratings in the ED. Moreover, numerous studies have shown that ML models can detect sepsis patients earlier than existing practices or traditional screening methods. The median lead time achieved by the ML alerts was significant in certain instances. Bedoya et al. [11] indicated a median detection lead time of around five hours before clinical recognition of sepsis, signifying that the model's warning criteria were satisfied hours before physicians usually diagnosed the condition. Wardi et al.'s [2] approach effectively predicted the progression of patients to septic shock 8-12 hours before onset by analyzing patterns in vital signs and laboratory results. Operationally, models concentrating on triage data successfully identified high-risk patients upon their arrival at the ED. Horng et al. [1] demonstrated that the inclusion of triage nurse free-text notes enhanced early sepsis prediction upon patient arrival. Brann et al. [8] explicitly assessed the system's efficacy in nurse-initiated sepsis screening during triage: the ML model would have detected 76% of the sepsis cases overlooked by triage nurses, while accurately identifying all instances recognized by the nurses. Similarly, Prasad et al. [5] discovered that their triage-based logistic model could more rapidly stratify patient risk compared to standard treatment, delivering an immediate risk score that surpassed qSOFA during the initial assessment in the ED. The capacity to identify sepsis at an earlier stage (either before shock or at the onset of an ED visit) represents a significant benefit of ML-driven systems, potentially facilitating earlier interventions than would often occur under conventional protocols.

Clinical Implementation Outcomes

Only three of the 11 studies extended beyond diagnostic metrics to evaluate real-world clinical outcomes resulting from the application of ML sepsis alerts. The studies included a quasi-experimental before-and-after analysis of a deployed deep learning alert, a prospective multi-center cohort study assessing an integrated alert system (TREWS) in routine care, and a single-center cluster-randomized trial comparing an ML alert to conventional treatment. Conversely, the other research focused on model creation and validation, lacking reports on patient outcomes or alterations in care processes. In the implementation trials, deploying ML-based early warning systems in the ED was associated with improvements in sepsis treatment process metrics and, in two instances, significant benefits in patient outcomes. The objective of early sepsis warnings is to accelerate therapy, including the timely introduction of antibiotics. The cluster-randomized trial in Thailand revealed a significant enhancement in the prompt administration of antibiotics: 68% of sepsis patients in the ML alert group received medicines within one hour of ED presentation, in contrast to around 60% in the standard care group, representing an absolute increase of approximately 8.3%. Three hours post-arrival, antibiotic administration in the ML group exceeded that of the control group by approximately 5.5%. The acceleration in therapy was both statistically significant and clinically meaningful in the trial. In the multihospital TREWS deployment, instances in which the ED physicians recognized and responded to the ML alarm saw a more rapid beginning of treatment on average compared to cases with no alert or reaction. In addition to antibiotics, compliance with sepsis management procedures (bundles) is enhanced under ML alarm systems. In the COMPOSER before-after research, adherence to the three-hour sepsis treatment bundle improved by around 5% absolute following the adoption of the ML alert. This indicates that a greater number of patients underwent all prescribed procedures (including fluid resuscitation, lactate assessment, and vasopressor administration where necessary) within the timeframe stipulated by the guidelines. Furthermore, the ML-driven methodology demonstrated enhanced accuracy in initiating sepsis care: the cluster randomized controlled trial (RCT) indicated a reduction in false activations of the sepsis protocol relative to the previous SIRS-based screening, signifying improved specificity in recognizing authentic sepsis cases that required the care bundle. In summary, ML early warning systems assisted ED teams in administering sepsis care more swiftly and consistently in accordance with protocol standards.

Mortality and Clinical Outcomes

Two studies indicated a decrease in mortality linked to the implementation of ML sepsis notifications. Boussina et al. [6] observed that following the deployment of their deep learning EHR alert (COMPOSER) in two EDs, in-hospital mortality for sepsis patients decreased from a baseline of 13.1% to 11.2% after implementation. The 1.9 percentage-point decline signifies a 17% relative reduction in mortality, indicating that the alert's prompt detection and ensuing management alterations had a quantifiable effect on survival. In the TREWS system research Adams et al. (2022) [7], the analysis of outcomes revealed that when physicians acted on the ML signal and confirmed sepsis, then initiated treatment, patient mortality was 3.3% lower in absolute terms compared to cases where the alert was disregarded or absent. The advantages in that trial were primarily evident in high-risk patients identified by the model, indicating that the alert was especially beneficial for the most critically ill category. In addition to survival, various clinical outcomes and surrogate markers improved with the utilization of ML alerts. The TREWS evaluation revealed that patients whose sepsis alarms were acknowledged and therapies initiated immediately had a reduced hospital length of stay (LOS) and diminished organ failure progression compared to identical patients for whom the alert was disregarded. The summary could not quantify precise decreases in length of stay; however, the trend indicated a preference for the alert-intervention group, implying a more efficient recovery process. Similarly, organ dysfunction scores (e.g., SOFA) were superior in the intervention group: Adams et al. [7] found markedly lower maximum organ failure ratings in patients managed with alertness. Boussina et al. [6] observed a modest yet significant reduction in organ dysfunction 72 hours following ED admission, with a mean SOFA score change at 72 hours being 4% lower after alert implementation, suggesting a slight improvement in organ function during the initial hospitalization period. Although the study did not reveal a significant change in overall hospital length of stay, the decrease in SOFA trajectory aligns with improved early care. Importantly, none of the studies indicated any detrimental effects from the implementation of ML alerts (such as alert fatigue resulting in poorer outcomes); rather, the existing data imply that when thoughtfully incorporated into ED workflows, ML-based early warning systems can enhance care without apparent negative consequences. 

In conclusion, the findings from these 11 studies demonstrate that ML early warning systems for sepsis in the ED are useful in two primary aspects:

(1) Diagnostic precision and prompt identification: They regularly surpassed conventional screening methods in detecting sepsis, frequently offering earlier alerts (several hours before standard recognition or during patient triage) with high sensitivity and satisfactory specificity.

(2) Clinical implications upon implementation: In trials that implemented these systems, the ML warnings resulted in expedited treatment (enhancing the timeliness of antibiotic administration and adherence to care bundles) and were correlated with improved patient outcomes, including diminished organ failure, reduced hospital stays, and decreased mortality rates.

The findings emphasize the capability of ML-based sepsis warnings to markedly improve early sepsis management in EDs, while also indicating the necessity to address false positives and to assess the performance of each model within the local clinical context.

## Review

The global burden of sepsis is still quite high [1], despite advancements in its care, and early detection in the ED is notoriously challenging because of its subtle and varied presentation [2]. According to our comprehensive study, when compared to traditional screening methods, ML-based early warning systems can greatly increase the timely diagnosis of sepsis in ED patients. In terms of predicted accuracy, ML models routinely beat conventional metrics like SIRS and qSOFA in the included trials [3]. On the same cohort, for example, a retrospective random forest model had an AUROC of approximately 0.93, which was much higher than that of SIRS (~0.81) or qSOFA (~0.56) [4]. Similarly, in external validation, a logistic *qSOFA-sepsis* model without laboratory data demonstrated much superior discrimination than qSOFA (AUROC 0.766 vs. 0.579) [5]. These results highlight the potential of data-driven methods to identify individuals who simpler scores miss [7] and reflect the low sensitivity of fast SOFA shown in earlier ED investigations [6]. The capacity of ML systems to identify sepsis before clinical judgment or conventional methods is a significant benefit. According to our analysis, ML notifications frequently arrive several hours before clinicians are aware of them. An ensemble model by Wardi et al. [2] predicted progression to septic shock 8-12 hours ahead of time [9], and a deep learning model by Bedoya et al. [11] warned a median of four to five hours before traditional criteria would trigger [8]. Notably, high-risk patients can be flagged by ML-based triage technologies as soon as they arrive at the ED. Horng et al. [1] showed that sepsis could be predicted upon presentation by integrating triage nurse free text into an ML model [10]. In addition to identifying every case that nurses did notice, an NLP-driven triage model would have identified 76% of sepsis cases that were overlooked by standard nurse screening [11]. Similarly, an instantaneous risk score that stratified patients more quickly and accurately than the qSOFA bedside score during initial assessment was generated by a straightforward logistic regression model that was available at triage (the *attention bias* model by Prasad et al. [5]) [12]. These findings demonstrate that, in comparison to traditional methods, ML-based warnings that make use of extensive ED data and sophisticated pattern recognition can significantly speed up the detection of sepsis in the ED. However, specificity is sometimes sacrificed for increased sensitivity. The included studies only reported small PPVs for their ML warnings; for instance, the TREWS alert produced ~20% PPV with ~82% sensitivity in a multi-center research [13], and the triage NLP model had a PPV of ~18% [14]. This indicates that a large number of alarms were false positives, a trade-off acknowledged when sensitivity is prioritized in calibrating sepsis models. One acknowledged issue with sepsis early warning systems is high false alarm rates [15]. For comparison, the popular MEWS/qSOFA-based screening frequently has similarly low PPVs and can miss more patients [16]. However, the alert load seen here (about four to five false alarms per actual sepsis case) is on par with or better than that of traditional screening. The fact that no study has found any evidence of alert fatigue or other negative effects from using the ML systems is a significant finding. These ML alerts did not seem to overwhelm or divert providers with unnecessary alarms when they were introduced carefully [17]. Indeed, there was no evidence of adverse effects, and practitioners typically responded to alarms by assessing and treating patients earlier [18]. Conversely, in several studies, the implementation of ML sepsis alarms was linked to better clinical outcomes and results. Early sepsis therapy metrics improved in half of the included trials that used the ML model in practice. According to the cluster-randomized experiment by Kijpaisalratana et al. [9], for instance, the percentage of patients who received antibiotics within an hour of triage increased to 68% when they received a real-time ML alert, as opposed to 60% when they received standard treatment [19]. Another study showed that an ML-based pharmacist-clinician alert workflow reduced the time to antibiotics and enhanced 28-day outcomes [20]. The majority of the studies in our study were retrospective, single-center assessments [21], which carry a risk of bias in estimating model performance [22], despite the encouraging nature of these outcome improvements. Few studies employed prospective, blinded, or randomized designs, and sample sizes and sepsis prevalence varied greatly. Stronger interventional evidence is undoubtedly needed, given that only two randomized controlled studies have thoroughly evaluated patient outcomes directly linked to ML-based sepsis alarms [19,20]. The application of ML models in actual emergencies is another significant obstacle. Alert tiredness is still a major issue, particularly in EDs with large patient volumes. Clinicians were greatly dissatisfied with models such as the Epic Sepsis Model because they missed the majority of cases of genuine sepsis while generating hundreds of alerts [23]. The possibility of flooding providers with false positives was highlighted by the PPV, which was less than 10% [24]. High-sensitivity models may make this problem worse. Therefore, careful interface design and efficient tweaking are essential [25]. Additionally, clinical confidence in ML suggestions is still low. Without knowing how the *black box *predictions were made, many doctors are reluctant to act on them [26]. According to studies, interpretable ML techniques that clarify the driving characteristics and support clinical intuition are more likely to be employed [27,28]. The goal of recent work in explainable AI (XAI) frameworks like SHAP (SHapley Additive exPlanations) and LIME (Local Interpretable Model-agnostic Explanations) has been to increase the transparency of predictions [29]. Adding a layer of interpretability enhanced physician engagement with the system in one real-world implementation [30]. Additionally, it seems that incorporating notifications within current processes rather than introducing intrusive pop-ups improves compliance [31]. Another enduring constraint is generalizability. Due to variations in practice patterns, patient demographics, and EHR infrastructure, models developed on data from a single institution frequently perform poorly when used in various contexts [32]. Numerous sepsis ML studies continue to underreport external validation [33]. Only 15% of published sepsis prediction models had been verified outside of their originating institution, according to one meta-analysis [34]. This hinders broad adoption and emphasizes the necessity of consistent benchmarking and cooperative, multi-center datasets [35]. Fairness and bias must be addressed as well. Existing discrepancies can be encoded by ML models trained on skewed datasets; for instance, they may perform poorly on underrepresented ethnic or socioeconomic groups [36]. In an attempt to evaluate these issues, recent research has discovered quantifiable performance differences between demographic subgroups in a number of sepsis models [37]. Therefore, regular subgroup analysis, bias reduction techniques, and fairness reporting frameworks are necessary for ethical deployment [38]. Despite these obstacles, there are positive lessons to be learned from successful ML integration in EDs. ML warnings have enhanced sepsis management when paired with bundled care workflows or protocolized response teams [39]. In the TREWS system, lower mortality was linked to provider responsiveness to the alarm (as opposed to the alert alone) [14]. It seems that these systems function best when they are integrated into a therapeutic setting that is encouraging and offers training, feedback, and multidisciplinary ownership [40]. From a policy perspective, ML techniques help with ongoing initiatives to raise the standard and quality of sepsis care. AI-enabled alerts could aid in achieving the time to antibiotics and bundle compliance metrics that are being used to evaluate institutions more and more [41]. However, due to a lack of prospective outcome evidence, professional societies such as the Surviving Sepsis Campaign and the Society of Critical Care Medicine (SCCM) have not yet publicly recommended ML systems [42]. According to preliminary research, ML-based alerts could lower expenses by avoiding intensive care unit admissions or reducing hospital stays [43]. The need for early detection was further supported by an investigation that found that sepsis mortality rose by 7.6% for every hour if adequate care was delayed [44]. However, there are still a few official cost-effectiveness assessments [45]. Several priorities should be the focus of future research. Initially, more extensive, prospective studies in many contexts are required to verify that ML warnings enhance tangible results, such as mortality, rather than merely procedures [46]. Second, before deployment, external validation and recalibration techniques need to be standardized [47]. Models may be able to adjust to local data with the aid of transfer learning and federated learning without sacrificing privacy [48]. Third, scientists need to keep creating interpretable models and researching how physicians follow, disregard, or trust their advice [49]. An interesting option is the use of hybrid models - combining clinical judgment with ML risk scores - which may outperform either alone [50]. Phenotype-specific sepsis prediction is another developing field that uses ML to customize alarms to different biological subtypes [51]. Furthermore, to guarantee accessibility, equity, and trust, the developing field of human-centered AI places a strong emphasis on developing tools in collaboration with frontline users [52]. Lastly, health systems will need governance frameworks for algorithm review, monitoring, and accountability as AI rules develop [53]. Transparent communication with patients and clinicians regarding the use of AI in healthcare decision-making, user feedback loops, and ongoing model performance tracking are all examples of best practices [54,55]. Institutions may consider developing AI oversight committees - analogous to ethics boards - to examine new tools and ensure responsible integration [56]. To sum up, ML-based sepsis early warning systems present a revolutionary chance to enhance emergency care. These models perform better than conventional screening methods, identify sepsis sooner, and occasionally lead to better clinical results. However, additional validation, equality protections, and careful implementation tactics are necessary to reach their full potential. ML-driven warnings could be a key component of contemporary sepsis management in the ED with the correct infrastructure, regulatory, and user-centered design investments [57-62].

## Conclusions

ML-based sepsis alerts show superior diagnostic accuracy compared to traditional rule-based tools, with many models providing earlier detection of sepsis in emergency department settings. Early evidence suggests many clinical benefits, resulting in improved treatment timeliness, such as timely antibiotic administration and better adherence to sepsis care bundles, which translates into better treatment outcomes, including reduction in organ failure, hospital stay, and mortality. Nevertheless, the overall strength of evidence is limited because most of the studies were retrospective single-center studies, and the RCTs have not yet been conducted on a large scale. Future research should focus on conducting multi-center prospective trials to enhance the assessment of both the diagnostic performance and patient outcomes.

Further prospective validation and thoughtful integration are essential to realize ML-based sepsis alerts' full potential. Although current research presents encouraging outcomes, the regular clinical incorporation of ML models necessitates the resolution of practical obstacles, including provider acceptance, technical integration with EHR systems, and the reduction of false alarms. Future work must emphasize explainability and stringent prospective validation across varied therapeutic environments to ascertain value.
